# Evaluation of the VITEK2 AST-XN17 card for the detection of carbapenemase-producing *Enterobacterales* in isolates primarily producing metallo β-lactamase

**DOI:** 10.1007/s10096-022-04424-5

**Published:** 2022-02-24

**Authors:** Tomokazu Kuchibiro, Masaru Komatsu, Katsutoshi Yamasaki, Tatsuya Nakamura, Makoto Niki, Hisaaki Nishio, Kaneyuki Kida, Masanobu Ohama, Akihiro Nakamura, Isao Nishi

**Affiliations:** 1 Department of Clinical Laboratory, Naga Municipal Hospital, 1282 Uchita, Kinokawa, Wakayama, 649-6414 Japan; 2 Department of Clinical Laboratory Science, Tenri Health Care University, Nara, Japan; 3 Department of Medical Life Science, Kurashiki University of Science and the Arts, Okayama, Japan; 4 Department of Medical Technology and Sciences Facility of Health Sciences, Kyoto Tachibana University, Kyoto, Japan; 5 Department of Infection Control and Prevention, Osaka City University Hospital, Osaka, Japan; 6 Department of Clinical Laboratory, Shiga Medical Center for Children, Shiga, Japan; 7 Clinical Laboratory, Japanese Red Cross Otsu Hospital, Shiga, Japan; 8 Laboratory for Clinical Investigation, Osaka University Hospital, Osaka, Japan

**Keywords:** Carbapenemase-producing *Enterobacterales*, Antimicrobial susceptibility testing, VITEK2, Advance Expert System

## Abstract

Carbapenemase-producing *Enterobacterales* (CPE) are not always resistant to carbapenem antimicrobial susceptibility testing (AST) and can be difficult to detect. With the newly created VITEK2 AST-XN17 card, the types of antibiotics measured in AST can be increased. In this study, we evaluated the detectability of CPE using the results of AST with multiple antimicrobial agents with additional measurements of the AST-XN17 card. In addition, we evaluated the CPE detectability of comments on CPE using the VITEK2 Advance Expert System (AES). In total, 169 Enterobacterales samples, including 76 non-CPE and 93 CPE, collected from multiple medical institutions in the Kinki region of Japan, were used in this investigation. AST with VITEK2 was performed by adding the AST-XN17 card in addition to the AST-N268 or AST-N404 card. Measurement results were identified using cutoff values, primarily Clinical and Laboratory Standards Institute breakpoints, and the CPE detection capability of each antibiotic was evaluated in several terms, including sensitivity and specificity. The drugs highly sensitive to CPE detection were faropenem (FRPM) > 2 µg/mL at 100% and meropenem > 0.25 µg/mL at 98.9%; the highest specificity to CPE detection was for avibactam/ceftazidime (AVI/CAZ) > 8 µg/mL at 100%. The sensitivity and specificity of each card in the AES output were 86.2% and 94.7% for AST-N404 and AST-XN17 and 91.5% and 90.8% for AST-N268 and AST-XN17, respectively. AST using the VITEK2 AST-XN17 card is a useful test method of screening for CPE.

## Introduction

Carbapenems are a commonly used primary therapeutic option for serious infections caused by gram-negative bacilli. They are often considered agents of last resort. In recent years, an increase in carbapenemase-producing *Enterobacterales* (CPE) has been reported, and this has become a global problem [1, 2]. Resistance to carbapenem among *Enterobacterales* is mediated by various mechanisms, including production of carbapenem-hydrolyzing enzymes (so-called carbapenemases), alteration in outer membrane permeability, and in certain circumstances, overproduction of an AmpC- or extended-spectrum β-lactamase (ESBL)-type enzyme combined with porin loss/modification [1, 3–5]. Carbapenemase is classified as metallo-β-lactamase (MBL) and serine carbapenemase, and several types of carbapenemase-producing genes have been reported in *Enterobacterales* [6]. As there are various types of carbapenemase-producing genes and *Enterobacterales* species, they show different patterns of antibiotic resistance [6]. Among CPE, there are so-called stealth-type strains in which the minimum inhibitory concentrations (MIC) of carbapenem are not determined to be resistant beyond the breakpoint upon antimicrobial susceptibility testing (AST) [7]. Therefore, it may be difficult to detect CPE based only on the results of routine AST such as with imipenem (IPM) and meropenem (MEPM) in case with low MIC values cases. In this study, a newly developed VITEK2 AST-XN17 card (bioMérieux, Marcy-l'Étoile, France) was used. This card allows users to perform AST for the measurement of various antibiotics in addition to the standard antibiotics tested by the existing VITEK2 AST card; thus, susceptibility measurement of a wider variety of antibiotics in addition to the AST card used for routine measurement becomes possible. The present study aimed to evaluate the detectability of CPE based on the results of AST with multiple antimicrobial agents in conjunction with additional measurements of the AST-XN17 card.

## Materials and methods

The strains evaluated were stock and clinical isolates of β-lactamase-producing *Enterobacterales* strains collected from 2010 to 2020 by the Naga Municipal Hospital and the Study of Bacterial Resistance in the Kinki Region of Japan. Isolates were obtained from various clinical sources (e.g., blood cultures, urine, and sputum), and there was no duplication of isolates from the same patient. A total of 169 *Enterobacterales* isolates, including 76 non-CPE (including extended-spectrum β-lactamases and/or AmpC producers) and 93 CPE (79 IMP, 2 VIM, 1 KPC, 2 NDM, 2 OXA, 6 GES, and 1 IMP/GES-producing *Enterobacterales*) isolates collected from multiple medical institutions in the Kinki region of Japan, were used in this investigation. Confirmation of resistance mechanisms was determined via PCR and sequence analysis, as previously reported [8–12]. The targeted carbapenemase genes were *bla*GES-like, *bla*IMP-1-like, *bla*IMP-2-like, *bla*KPC-like, *bla*oxa-48-like, *bla*NDM-like, and *bla*VIM-like. The amplified products were sequenced using an automated DNA sequencer (ABI 3100, Applied Biosystems, Foster City, CA). In the group containing non-CPE, the PCR results were negative for carbapenemase, and strains suspected of overexpression of chromosomal AmpC or ESBL and/or plasmid-mediated AmpC-producing strains were used. Non-CPE strains were confirmed to be negative by performing phenotypic tests by PCR of the carbapenemase gene and modified carbapenem inactivation method test (mCIM) according to the Clinical and Laboratory Standards Institute (CLSI) M100-S29 [13].

The AST of the test strain was performed using VITEK2 Compact (bioMérieux), and the operation procedure was conducted according to the VITEK2 instruction manual.

The software used was VITEK2 version 9.02, and CLSI M100-S29 [13] was used as the judgment breakpoint. For the test strain, AST by VITEK2 was performed using two types of routine cards, AST-N404 and AST-N268, with AST-XN17 used as an additional card. For MIC values, IPM, MEPM, and TAZ/PIPC were measured with AST-N404, and IPM and MEPM were measured with AST-N268. With the AST-XN17 card, MIC values were measured for faropenem (FRPM), cefoxitin (CFX), ertapenem, latamoxef (LMOX), tazobactam/ceftolozane, and avibactam/ceftazidime (AVI/CAZ). Because AST-N404 and AST-N268 have significantly different algorithms called drug versions for drug measurement in IPM, measurements were performed using both types of cards in IPM and MEPM. If the measurement result was expressed as terminated results and the MIC value could not be calculated, it was judged to be indeterminate and excluded from the aggregation.

The cutoff value of each antibiotic conformed to CLSIM100-S29, and the MIC value of the breakpoints judged to be susceptibility was set as the cutoff value. The judgment was negative when the value was below the cutoff value and positive when the value was above the cutoff value. As a breakpoint for FRPM has not been set in CLSI, MIC 2 µg/mL was set independently as a cutoff value based on previously reported values [14, 15]. For MEPM, in addition to the CLSI breakpoint, the cutoff value was set to > 0.25 µg/mL, which is the closest to the EUCAST epidemiological cutoff value [16] in the VITEK2 measurement range. We defined sensitivity, specificity, positive predictive value, and negative predictive value of individual antibiotics to detect CPE using CLSI breakpoint-based cutoff values. Additionally, we created an algorithm for detecting CPE by combining screening with multiple antibacterial agents based on the results of AST.

For IPM and MEPM, we measured the MIC value by the broth microdilution method using the dry plate Eiken (Eiken Chemical, Tokyo). Subsequently, we assessed the sensitivity and specificity of the recommendations from the Advanced Expert System (AES) to detect CPE. We used AES recommendations expressed by two combinations, one of the AST-N404 and AST-XN17 cards and the other with the AST-N268 and AST-XN17 cards, and we evaluated each combination.

## Results

The MIC values of each antibiotic measured by VITEK2 are listed in Tables 1 and 2 for CPE and non-CPE isolates, respectively. Table 3 presents the results of CPE detection performance using the sensitivity, specificity, positive predictive value, and negative predictive value of each antibiotic and the evaluation of AES. The highly sensitive antibiotics were FRPM > 2 µg/mL at 100%, followed by MEPM > 0.25 µg/mL and CFX > 8 µg/mL at 98.9%, and the highest specificity was observed for AVI/CAZ > 8 µg/mL at 100% and MEPM 1 µg/mL at 96.2%.

**Table 1:** Results of minimum inhibitory concentrations (MICs) by VITEK2 in 93 carbapenemase-producing *Enterobacterales* isolates

Species	β-lactamase content		No. of strains	MICs(μg/mL) of BMD		AST-N404		AST-N268		XN17						
	Carbapenemase	ESBLs/PABL		IPM	MEPM	IPM	MEPM	IPM	MEPM	FRPM	ETPM	CFX	LMOX	TAZ/PIPC	TAZ/CTLZ	AVI/CAZ
*C. freundii* [8]	IMP-1		2	4	16	4 to ≥ 16	≥ 16	8 to ≥ 16	≥ 16	≥ 8	≥ 8	≥ 64	≥ 64	≥ 128	16	≥ 16
	IMP-6	CTX-M-2	4	≤ 0.5 to 2	4 to > 16	1	8 to ≥ 16	0.5 to 4	8 to ≥ 16	≥ 8	1 to ≥ 8	≥ 64	≥ 64	≤ 4	2 to 8	2 to ≥ 16
	IMP-19		1	> 16	> 16	8	≥ 16	8	≥ 16	≥ 8	1	≥ 64	≥ 64	≥ 128	16	≤ 0.12
	VIM-2		1	2	≤ 0.5	1	4	2	4	≥ 8	≥ 8	≥ 64	≥ 64	≥ 128	16	≥ 16
*E. cloacae* [7]	IMP-1		4	1 to 2	2 to 8	1 to 8	≥ 16	8 to ≥ 16	≥ 16	≥ 8	1 to ≥ 8	≥ 64	≥ 64	64 to ≥ 128	16 to ≥ 32	≥ 16
	IMP-1, GES-4		1	8	16	8	≥ 16	≥ 16	≥ 16	≥ 8	≥ 8	≥ 64	≥ 64	≥ 128	16	≥ 16
	IMP-6	CTX-M-2	1	1	> 16	0.5	≥ 16	≤ 0.25	≥ 16	≥ 8	≥ 8	≥ 64	≥ 64	16	≥ 32	≥ 16
	VIM-2		1	1	1	≥ 16	≥ 16	≥ 16	≥ 16	≥ 8	≥ 8	≥ 64	≥ 64	8	≥ 32	≥ 16
*E. coli* [17]	IMP-6		1	2	> 16	1	≥ 16	≤ 0.25	≥ 16	≥ 8	≥ 8	≥ 64	≥ 64	≤ 4	≥ 32	≥ 16
	IMP-6	CTX-M-2	12	≤ 0.5	≤ 0.5 to > 16	≤ 0.25 to 4	≥ 16	≤ 0.25 to 4	≥ 16	4 to ≥ 8	0.25 to ≥ 8	≥ 64	≥ 64	≤ 4 to 32	4 to ≥ 32	2 to ≥ 16
	IMP-6	CTX-M-2, -M-14	2	1	> 16	≤ 0.25	≥ 16	≤ 0.25	≥ 16	≥ 8	2 to ≥ 8	≥ 64	≥ 64	≤ 4	8	≥ 16
	IMP-6	CTX-M-2, -M-27	1	1	16	1	≥ 16	2	≥ 16	≥ 8	≥ 8	≥ 64	≥ 64	≤ 4	≥ 32	≥ 16
	NDM-5	CTX-M-65	1	8	> 16	≥ 16	≥ 16	≥ 16	≥ 16	≥ 8	≥ 8	≥ 64	≥ 64	≥ 128	≥ 32	≥ 16
*K. aerogenes* [1]	IMP-6	CTX-M-2	1	≤ 0.5	> 16	4	≥ 16	4	≥ 16	≥ 8	≥ 8	≥ 64	≥ 64	≥ 128	≥ 32	≥ 16
*K. oxytoca* [4]	IMP-6	CTX-M-2	1	≤ 0.5	> 16	≤ 0.25	≥ 16	≤ 0.25	≥ 16	≥ 8	≥ 8	≥ 64	≥ 64	16	≥ 32	≥ 16
	IMP-34		3	≤ 0.5	4 to > 16	≤ 0.25 to 2	≥ 16	≤ 0.25 to 2	≥ 16	≥ 8	≥ 8	≥ 64	≥ 64	≤ 4 to 16	8 to ≥ 32	8 to ≥ 16
*K. pneumoniae* (50)	GES-4		4	≤ 0.5 to 1	≤ 0.5 to 1	2 to 4	1	2 to ≥ 16	1 to 4	≥ 8	0.25 to 2	≥ 64	≤ 4 to 8	64 to ≥ 128	8 to ≥ 32	0.25 to 1
	GES-4	CTX-M-15	1	1	1	4	1	4	4	≥ 8	2	≥ 64	8	≥ 128	≥ 32	1
	IMP-1		2	≤ 0.5 to 4	4 to 8	8 to ≥ 16	≥ 16	8 to ≥ 16	≥ 16	≥ 8	≥ 8	≥ 64	≥ 64	8 to ≥ 128	≥ 32	≥ 16
	IMP-6		1	2	8	8	≥ 16	≥ 16	≥ 16	≥ 8	≥ 8	≥ 64	≥ 64	64	≥ 32	≥ 16
	IMP-6	CTX-M-2	34	≤ 0.5 to 2	≤ 0.5 to > 16	≤ 0.25 to 4	≤ 0.25 to ≥ 16	≤ 0.25 to 2	≤ 0.25 to ≥ 16	4 to ≥ 8	0.25 to ≥ 8	32 to ≥ 64	16 to ≥ 64	≤ 4 to ≥ 128	2 to ≥ 32	1 to ≥ 16
	IMP-6	CTX-M-2, DHA	1	1	> 16	2	≥ 16	2	≥ 16	≥ 8	≥ 8	≥ 64	≥ 64	32	8	≥ 16
	IMP-8		1	8	8	≥ 16	4	≥ 16	4	≥ 8	≥ 8	≥ 64	≥ 64	≥ 128	≥ 32	≥ 16
	IMP-19	CTX-M-1	1	0.5	0.25	4	4	≥ 16	4	≥ 8	0.25	≥ 64	≥ 64	8	≥ 32	≥ 16
	IMP-19	CTX-M-3	1	1	0.25	4	1	≥ 16	1	≥ 8	1	≥ 64	≥ 64	16	≥ 32	≥ 16
	IMP-34		1	1	2	8	≥ 16	≥ 16	≥ 16	≥ 8	2	≥ 64	≥ 64	32	≥ 32	≥ 16
	KPC-2	CTX-M-14	1	> 16	> 16	≥ 16	≥ 16	≥ 16	≥ 16	≥ 8	≥ 8	≥ 64	≥ 64	≥ 128	≥ 32	1
	OXA-48		1	1	0.5	4	4	4	2	≥ 8	≥ 8	≤ 4	16	≥ 128	1	0.25
	OXA-181		1	2	1	2	1	2	2	≥ 8	≥ 8	16	16	≥ 128	≥ 32	0.5
*M. morganii* [1]	IMP-1		1	8	16	≥ 16	4	1	4	≥ 8	0.5	≥ 64	≥ 64	≤ 4	8	≥ 16
*P. mirabilis* [1]	NDM-1	CTX-M-65	1	8	4	≥ 16	≥ 16	≥ 16	≥ 16	≥ 8	≥ 8	32	16	8	≥ 32	≥ 16
*P. rettgeri* [1]	IMP-1		1	16	16	≥ 16	≥ 16	≥ 16	≥ 16	≥ 8	≥ 8	≥ 64	≥ 64	8	16	2
*S. marcescens* [3]	GES-5		1	8	> 16	8	≥ 16	8	≥ 16	≥ 8	≥ 8	≥ 64	≥ 64	≥ 128	8	2
	IMP-1		2	16 to > 16	16 to > 16	8 to ≥ 16	≥ 16	≥ 16	≥ 16	≥ 8	≥ 8	≥ 64	≥ 64	64 to ≥ 128	16 to ≥ 32	≥ 16

*ESBLs* extended-spectrum β-lactamases, *PABL* plasmid mediated AmpC β-lactamase, *BMD* broth microdilution method, *IPM* imipenem, *MEPM* meropenem, *FRPM* faropenem, *ETPM* ertapenem, *LMOX* latamoxef, *TAZ/PIPC* tazobactam/piperacillin, *TAZ/CTLZ* tazobactam/ceftolozane, *AVI/CAZ* avibactam/ceftazidime

**Table 2:** Results of minimum inhibitory concentrations (MICs) by VITEK2 in 76 non-carbapenemase-producing *Enterobacterales* isolates

Species	β-lactamase content		No. of strains	MICs( μg/mL) of BMD		AST-N404		AST-N268		XN-17						
	ESBLs	PABL		IPM	MEPM	IPM	MEPM	IPM	MEPM	FRPM	ETPM	CFX	LMOX	TAZ/PIPC	CTLZ/TAZ	AVI/CAZ
*E. cloacae* [2]		C-AmpC	2	2–4	≤ 0.5 to 2	1	≤ 0.25	2	≤ 0.25	2 to 4	≤ 0.12	≥ 64	≤ 4	≤ 4	≤ 0.25	≤ 0.12 to 0.5
*E. coli* (38)	CTX-M-1		4	≤ 0.5	≤ 0.5	≤ 0.25	≤ 0.25	≤ 0.25	≤ 0.25	≤ 0.5 to 1	≤ 0.12	≤ 4	≤ 4	≤ 4	≤ 0.25 to 16	≤ 0.12
	CTX-M-2		4	≤ 0.5	≤ 0.5	≤ 0.25	≤ 0.25	≤ 0.25	≤ 0.25	≤ 0.5 to 2	≤ 0.12 to 0.25	≤ 4 to 16	≤ 4	≤ 4 to ≥ 128	2 to 16	≤ 0.12 to 0.5
	CTX-M-9		4	≤ 0.5	≤ 0.5	≤ 0.25	≤ 0.25	≤ 0.25	≤ 0.25	1	≤ 0.12	≤ 4	≤ 4	≤ 4 to 16	≤ 0.25	≤ 0.12
	SHV-12		5	≤ 0.5	≤ 0.5	≤ 0.25	≤ 0.25	≤ 0.25	≤ 0.25	≤ 0.5 to 1	≤ 0.12	≤ 4 to 16	≤ 4	≤ 4	≤ 0.25	≤ 0.12
	TEM-20		1	≤ 0.5	≤ 0.5	0.5	≤ 0.25	≤ 0.25	≤ 0.25	2	0.5	8	8	≥ 128	4	0.25
	TEM-52		1	≤ 0.5	≤ 0.5	≤ 0.25	≤ 0.25	≤ 0.25	≤ 0.25	1	≤ 0.12	≤ 4	≤ 4	≤ 4	≤ 0.25	≤ 0.12
		CIT	8	≤ 0.5	≤ 0.5	≤ 0.25 to 1	≤ 0.25	≤ 0.25 to 0.5	≤ 0.25	1 to ≥ 8	≤ 0.12 to 0.25	32 to ≥ 64	≤ 4 to 8	≤ 4 to 64	≤ 0.25 to 2	≤ 0.12 to 0.25
		DHA	3	≤ 0.5	≤ 0.5	≤ 0.25	≤ 0.25	≤ 0.25	≤ 0.25	2 to 4	≤ 0.12	≥ 64	≤ 4	≤ 4 to 32	≤ 0.25	≤ 0.12
		MOX	1	≤ 0.5	≤ 0.5	≤ 0.25	≤ 0.25	≤ 0.25	≤ 0.25	≥ 8	≤ 0.12	≥ 64	32	16	0.5	≤ 0.12
	CTX-M-1	CIT	5	≤ 0.5	≤ 0.5	≤ 0.25 to 1	≤ 0.25	≤ 0.25 to 1	≤ 0.25	≤ 0.5 to 1	≤ 0.12 to 0.25	≤ 4 to ≥ 64	≤ 4	≤ 4 to 16	≤ 0.25 to 1	≤ 0.12 to 0.25
	CTX-M-1,-M-9	CIT	1	≤ 0.5	≤ 0.5	≤ 0.25	≤ 0.25	≤ 0.25	≤ 0.25	1	≤ 0.12	16	≤ 4	8	1	0.25
	CTX-M-2	CIT	1	≤ 0.5	≤ 0.5	1	≤ 0.25	≤ 0.25	≤ 0.25	2	0.25	≥ 64	≤ 4	8	2	0.5
	CTX-M-9	CIT	2	≤ 0.5	≤ 0.5	≤ 0.25	≤ 0.25	≤ 0.25	≤ 0.25	2 to 4	0.25	≥ 64	≤ 4 to 16	32 to ≥ 128	4 to ≥ 32	≤ 0.12 to 0.5
	CTX-M-9	DHA	1	≤ 0.5	≤ 0.5	1	≤ 0.25	≤ 0.25	≤ 0.25	4	≤ 0.12	≥ 64	≤ 4	≤ 4	4	0.25
	SHV	CIT	1	≤ 0.5	≤ 0.5	1	≤ 0.25	≤ 0.25	≤ 0.25	1	≤ 0.12	≥ 64	≤ 4	16	1	0.25
*K. aerogenes* [3]		C-AmpC	3	2 to 8	1 to 4	4	1	2	≤ 0.25 to 4	≥ 8	≤ 0.12 to ≥ 8	≥ 64	≤ 4 to 32	≤ 4 to 32	≤ 0.25 to 8	≤ 0.12 to 2
*K. oxytoca* [4]		CIT	2	≤ 0.5	≤ 0.5	≤ 0.25 to 0.5	≤ 0.25	≤ 0.25 to 0.5	≤ 0.25	≤ 0.5 to 1	≤ 0.12	≤ 4 to 16	≤ 4	≤ 4	≤ 0.25	≤ 0.12
		DHA	1	≤ 0.5	≤ 0.5	2	≤ 0.25	≤ 0.25	≤ 0.25	≥ 8	1	≥ 64	≤ 4	≥ 128	16	0.5
		MOX	1	≤ 0.5	≤ 0.5	≤ 0.25	≤ 0.25	1	≤ 0.25	≥ 8	0.25	≥ 64	≥ 64	16	0.5	0.5
*K. pneumoniae* [19]	CTX-M-1		2	≤ 0.5	≤ 0.5	≤ 0.25	≤ 0.25	1	≤ 0.25	1 to 4	≤ 0.12 to 0.25	≤ 4	≤ 4	32 to ≥ 128	16 to ≥ 32	0.5
	CTX-M-2		1	≤ 0.5	≤ 0.5	0.5	≤ 0.25	≤ 0.25	≤ 0.25	2	≤ 0.12	8	≤ 4	≤ 4	≤ 0.25	≤ 0.12
	CTX-M-9		2	≤ 0.5	≤ 0.5	≤ 0.25	≤ 0.25	≤ 0.25	≤ 0.25	1 to 4	≤ 0.12	≤ 4	≤ 4	8 to ≥ 128	≤ 0.25	≤ 0.12
		CIT	3	≤ 0.5	≤ 0.5	2	≤ 0.25	0.5 to 1	≤ 0.25	≥ 8	0.25	≥ 64	8	8 to ≥ 128	≤ 0.25 to 8	0.25 to 0.5
		DHA	8	≤ 0.5 to 4	≤ 0.5 to 4	≤ 0.25 to 2	≤ 0.25 to 2	≤ 0.25 to 1	≤ 0.25 to 4	≥ 8	0.25 to ≥ 8	32 to ≥ 64	≤ 4 to ≥ 64	32 to ≥ 128	4 to ≥ 32	0.25 to 8
		MOX	1	≤ 0.5	≤ 0.5	≤ 0.25	≤ 0.25	1	≤ 0.25	≥ 8	0.5	≥ 64	≥ 64	16	2	1
	CTX-M-9	DHA	2	≤ 0.5	≤ 0.5	≤ 0.25 to 0.5	≤ 0.25	≤ 0.25	≤ 0.25	≥ 8	0.25	≥ 64	≤ 4	8	≤ 0.25 to 16	0.25 to 0.5
*P. mirabilis* [5]	CTX-M-2		3	≤ 0.5 to 1	≤ 0.5	2 to ≥ 16	1	2 to ≥ 16	1	≥ 8	≤ 0.12	8 to 32	≤ 4	≤ 4	≤ 0.25 to 8	≤ 0.12 to 0.25
		CIT	1	≤ 0.5	≤ 0.5	8	1	8	0.5	≥ 8	≤ 0.12	≥ 64	≤ 4	≤ 4	8	≤ 0.12
	CTX-M-2	CIT	1	≤ 0.5	≤ 0.5	4	≤ 0.25	2	≤ 0.25	≤ 0.5	≤ 0.12	32	8	≤ 4	0.5	≤ 0.12
*S. marcescens* [1]		C-AmpC	1	4	2	1	4	4	4	≥ 8	≥ 8	≥ 64	≥ 64	≥ 128	8	2

*ESBLs* extended-spectrum β-lactamases, *PABL* plasmid mediated AmpC β-lactamase, *BMD* broth microdilution method, *IPM* imipenem, *MEPM* meropenem, *FRPM* faropenem, *ETPM* ertapenem, *LMOX* latamoxef, *TAZ/PIPC* tazobactam/piperacillin, *TAZ/CTLZ* tazobactam/ceftolozane, *AVI/CAZ* avibactam/ceftazidime

**Table 3:** Evaluation of carbapenemase-producing *Enterobacterales* detection sensitivity and specificity for each drug and VITEK2 Advance Expert System

			CPE (n = 93)			non-CPE (n = 76)						
Card NO	Antimicrobial	Cut off value	Positive	Indeterminate	Negative	Positive	Indeterminate	Negative	Sensitivity (%)	Specificity (%)	PPV (%)	NPV (%)
AST-N404	IPM	1	47	0	46	12	3	61	50.5	83.6	79.7	57.0
	MEPM	1	85	0	8	3	0	73	91.4	96.2	96.6	90.1
	MEPM	0.25	92	0	1	7	0	69	98.9	90.8	92.9	98.6
AST-N268	IPM	1	45	0	48	11	0	65	48.4	85.5	80.4	57.5
	MEPM	1	88	0	5	3	0	73	94.6	96.1	96.7	93.6
	MEPM	0.25	92	0	1	8	0	68	98.9	89.5	92.0	98.6
AST-XN17	FRPM	2	93	0	0	33	0	43	100.0	56.6	73.8	100
	ETPM	0.5	84	0	9	6	0	70	90.3	92.1	93.3	88.6
	CFX	8	92	0	1	50	0	26	98.9	34.2	64.8	96.3
	LMOX	8	88	0	5	7	0	69	94.6	90.8	92.6	93.2
	TAZ/PIPC	16	60	0	33	20	2	54	64.5	73.0	75.0	62.1
	TAZ/CTLZ	2	90	0	3	21	2	53	96.8	71.6	81.1	94.6
	AVI/CAZ	8	65	0	28	0	2	74	69.9	100.0	100	72.5
Comments of AES												
AST-N404 + XN17			81		13	4		72	86.2	94.7	95.3	84.7
AST-N268 + XN17			86		8	7		69	91.5	90.8	92.5	89.6

*IPM* imipenem, *MEPM* meropenem, *FRPM* faropenem, *ETPM* ertapenem, *CFX* cefoxitin, *LMOX* latamoxef, *TAZ/PIPC* tazobactam/piperacillin, *TAZ/CTLZ* tazobactam/ceftolozane, *AVI/CAZ* avibactam/ceftazidime, *AES* VITEK2 Advance Expert System, *PPV* positive predictive value, *NPV* negative predictive value

## Discussion

The AST-XN17 card can measure additional antibiotics that have been reported to be useful as screening markers for CPE, such as FRPM and LMOX [18–20]. The antibiotic with the highest sensitivity was FRPM > 2 µg/mL, showing 100% sensitivity, as previously reported [19], and was considered to be very useful as a primary screening. However, because the specificity was as low as 56.6%, distinguishing CPE alone was challenging, and judging in combination with other agents seems highly useful. Screening for a combination of the most specific AVI/CAZ > 8 µg/mL and the most sensitive FRPM > 2 µg/mL yielded very useful results. If these results are obtained, the strain is likely an MBL-producing strain (65/65). Avibactam is a non-β-lactam–β-lactamase inhibitor that inhibits the activities of Ambler class A β-lactamases, including ESBLs and KPC, class C β-lactamases, and some class D β-lactamases [20]. The in vitro antibacterial activity of AVI/CAZ is likely to be effective against class A (including KPC) and class D (including OXA-48) CPE and AmpC and ESBL-producing *Enterobacterales* with porin loss/modification [20, 21].

However, AVI/CAZ is unlikely to show antibacterial activity in vitro because avibactam has no inhibitory effect on MBL. These characteristics of in vitro antibacterial activity of AVI/CAZ are similar to the results of this study and appear to be useful as specific markers for the differentiation between MBL and serine-carbapenemase.

Next to FRPM, MEPM > 0.25 µg/mL showed the highest sensitivity. In this study, we measured the MIC of MEPM using both the AST-N404 and AST-N268 cards; however, no difference in MIC values was observed between the two cards. The MIC values of IPM and MEPM obtained with VITEK2 tended to be higher in CPE than those obtained using the broth microdilution method. Therefore, it seems that the sensitivity of CPE screening at MEPM > 0.25 µg/mL was increased. When FRPM > 2 µg/mL and MEPM > 0.25 µg/mL were positive, the possibility of metallo-carbapenemase production was very high, similar to when AVI/CAZ > 2 µg/mL was positive, whereas the possibility of serine-carbapenemase production was suggested when AVI/CAZ > 2 µg/mL was negative. These results suggest that AVI / CAZ > 2 µg / mL may be a cutoff value that can classify MBL and serine-carbapenemase. Based on these results, it is possible to conduct CPE confirmation and genotyping tests. LMOX > 8 µg/mL showed a high positive/negative concordance rate and was considered useful for screening. Owing to the high specificity of LMOX, it may be useful to combine LMOX with FRPM. However, caution is required because the sensitivity to GES-type carbapenemase is low (1/6) and there is a tendency to show false positives for MOX-type plasmid-mediated AmpC β-lactamase.

**Fig. 1:**
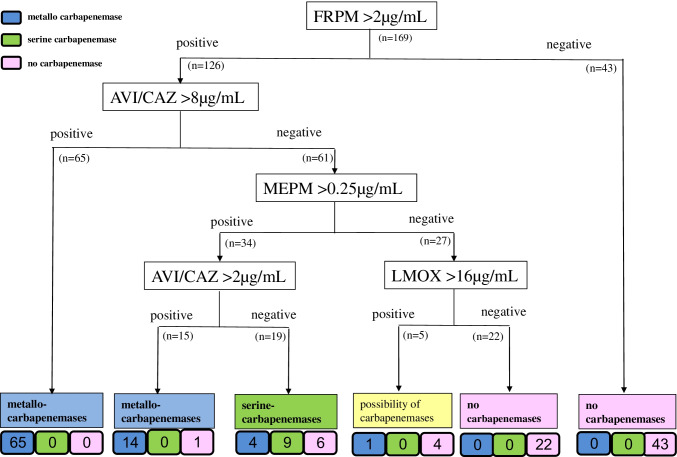
Algorithm for carbapenemase-producing *Enterobacterales* (CPE) screening via antimicrobial susceptibility testing using the AST-XN17 card of VITEK2

The algorithm for CPE screening using the AST results of VITEK2 is shown in Fig. 1. The results of this study may provide useful information for examining the necessity of conducting carbapenemase-detection tests. In addition, the recommendations of the VITEK2 AES for inferring carbapenemase production showed high detection performance for CPE with either card. The AES has been reported to perform well in the verification of AST results [22]. However, to the best of our knowledge, no studies evaluating the performance of CPE detection using the AES have been reported. This study is the first to evaluate the usefulness of the AES in CPE screening. The AES recommendation is useful for CPE screening, and if CPE are indicated, carbapenemase detection tests should be considered.

The test strains in this study included many strains collected in the Kinki area of Japan and included many IMP-6 (encoded by the *bla*IMP-6 gene) types. IMP-6, an IMP-type MBL, has been reported to confer a paradoxical IPM-susceptible but MEPM-resistant phenotype to *Enterobacterales* strains [23, 24]. Therefore, it is considered to be the cause of the bias that the sensitivity of IPM was evaluated as low in this study. In this study, we performed PCR to confirm the carbapenemase gene, which is frequently isolated, and did not perform whole genome sequencing. Therefore, the carbapenemase-negative group may carry a rare carbapenemase gene that was not targeted, which is a limitation of the present study. However, we consider it extremely unlikely, as negative results were obtained in phenotypic tests using mCIM. Furthermore, as the detection of serine-carbapenemase production such as KPC and OXA-48 is extremely rare in Japan, the small number of these strains examined should be taken into consideration in the interpretation of the results. The lack of detailed studies using serine-type carbapenemase-producing strains is a limitation of the present study and will be a future task.

In conclusion, CPE screening using the results of AST with the AST-XN17 card by VITEK2 appears to be a useful differential method. Establishing a method that does not overlook CPE is also extremely useful in the fields of antimicrobial stewardship and infection control.

## Data Availability

Not applicable.

## Abbreviations

AES: Advanced Expert System
AST: Antimicrobial susceptibility testing
CPE: Carbapenemase-producing Enterobacterales
IPM: Imipenem
MEPM: Meropenem
FRPM: Faropenem
AVI/CAZ: Avibactam/ceftazidime
MIC: Minimum inhibitory concentrations
CLSI: Clinical and Laboratory Standards Institute
MBL: Metallo-β-lactamase
ESBL: Extended-spectrum β-lactamases
mCIM: Modified carbapenem inactivation method test
LMOX: Latamoxef
